# Effective multi-functional biotechnological applications of protease/keratinase enzyme produced by new Egyptian isolate (*Laceyella sacchari* YNDH)

**DOI:** 10.1186/s43141-020-00037-7

**Published:** 2020-07-02

**Authors:** Doaa A. Goda, Ahmad R. Bassiouny, Nihad M. Abdel Monem, Nadia A. Soliman, Yasser R. Abdel Fattah

**Affiliations:** 1 Bioprocess Development Department, Genetic Engineering and Biotechnology Research Institute (GEBRI), City of Scientific Research and Technological Applications (SRTA-City), New Burg El-Arab City, Universities and Research Institutes Zone, Alexandria, Post 21934 Egypt; 2 Biochemistry Department, Faculty of Science, Alexandria, Egypt

**Keywords:** Protease, Keratinolytic activity, Experimental design, *Laceyella sacchari* YNDH

## Abstract

**Background:**

Due to a multitude of industrial applications of keratinolytic proteases, their demands are increasing. The present investigation studied the production and monitoring of the most possible multi-functional applications of YNDH thermoalkaline keratin-degrading enzyme.

**Results:**

This work is considered the first that reported YNDH strain closely related to *Laceyella sacchari* strain; YNDH is a producer of protease/keratinase enzyme and able to degrade natural keratin such as feathers, wool, human hairs, and nails. Experimental design Plackett-Burman (PBD) was applied to evaluate culture conditions affecting the production of thermoalkaline protease/keratinase. Afterwards, Box-Behnken design (BBD) was applied to find out the optimum level of significant variables namely, NH_4_Cl, yeast extract, and NaNO3 with a predicted activity of 1324.7 U/ml. Accordingly, the following medium composition and parameters were calculated to be optimum (%w/v): NH4Cl, 0.08; feather, 1; yeast extract, 0.04; MgSO_4_.7H_2_O, 0.02; NaNO_3_, 0.016; KH_2_PO_4_, 0.01; K_2_HPO_4,_ 0.01; pH, 8; inoculum size; 5%, cultivation temperature (Temp.) 45 °C and incubation time 48 h. The studied enzyme can degrade keratin-azure, remove proteinaceous materials, and is able to remove hairs from goat hides. These interesting characteristics make this enzyme a good candidate in many applications especially in detergent (Det.), in leather industries, and in pharmaceuticals particularly in nail treatment.

**Conclusion:**

The promising properties of the newly keratin-degrading protease enzyme from *Laceyella sacchari* strain YNDH would underpin its efficient exploitation in several industries to cope with the demands of worldwide enzyme markets.

## Background

Keratinolytic proteases (E.C 3.4.21/24/99.11) have substrate specificity where they have the ability to hydrolyze soluble protein such as casein, gelatin, and bovine serum albumin. In recent years, the demands of keratinolytic proteases are increasing due to their multitude in industrial applications such as the fertilizer, detergent (Det.), and textile industries [1]. For mature chickens, feathers account for up to 5% to 7% of their weight and are composed of over 90% crude protein, the main components being keratin and insoluble protein [2]. At present, feathers are converted to feather meal, a digestible dietary protein for animals, using physical and chemical treatments. These physicochemical conversion methods involve costly treatments under high-temperature (Temp.) and -pressure conditions in addition to the fact that the high Temp. causes the loss of a certain number of amino acids such as methionine, lysine, and tryptophan. Conventional disposal methods such as incineration of feathers have ecological disadvantages. Nowadays, feathers are converted into feather meal and used as animal feed supplement [3, 4]. However, the methods currently used for the conversion of feathers hydrolysis are chemical (alkaline hydrolysis) and the physical (steam pressure cooking); these methods, apart from being ecologically unfriendly and expensive due to the energetic cost, cause the loss of nutritionally essential amino acids [3–7].

Bacterial keratinolytic enzymes can have significant uses in biotechnological procedures involving keratin-containing waste from poultry and leather industries by developing non-polluting procedures. Insoluble feather keratins can be transformed to feature meal, fertilizers, and glues after enzymatic hydrolysis or used to produce rare serine, cysteine amino acids [3, 4, 8]. The elevated price of enzyme manufacturing is one of the main disadvantages faced by industrial enzyme manufacturers, as 30–40% of the manufacturing costs of many industrial enzymes are attributable to the price of the medium of cultivation [9]. For several centuries, statistical experimental designs have been used and can be implemented at different stages of an optimization strategy, such as screening tests or searching for ideal circumstances for targeted response(s) [10]. Recently, the findings evaluated through a statistically scheduled experiment are better recognized than the outcomes of the traditional one-variable-at-a-time (OVAT) test. The Plackett-Burman design (PBD) and response surface designs (RSD) are some of the common options when applying statistical models to bioprocessing [11–14]. The primary purpose of this research is to assess the circumstances of culture that affect the output of thermoalkaline protease with keratinolytic activity by locally isolated *Laceyella sacchari* YNDH and the possible application of this enzyme. In this way, the theme chosen for the application research can be exploited in many industries such as the leather industry, feeding, medical, and others.

## Methods

### Bacterial isolation

Samples were collected from different localities in Egypt (El-Minya governorate, Wadi El-Natrun lake, Hammam Pharaon) and Indonesia. For bacterial isolation, three different media were used, namely *alkaline nutrient* medium for Indonesian and El-Minya samples at 37 °C, pH 10.0 for 24 h, *Haloalkalophilic* medium for Wadi El-Natrun samples at 45 °C, pH 9.5 for 48 h and *Luria-Bertani liquid* medium (LB), for Hammam Pharaon samples at 55–70 °C, pH 7 for 24 h. After the cultivation time, 1 ml from each flask serially was diluted, and then about 100 μl of 10^−3^ and 10^−8^ dilutions were spread on Petri dishes and kept under the same isolation-cultural conditions for purification. The pure separate colonies were maintained on slants at 4 °C, and then sub-cultured regularly.

### Qualitative screening for protease activity

The qualitative screening was carried out using the same isolation medium but supplemented with 1% gelatin/casein as a substrate. The cultured plates were incubated for 24 h each at its own specific Temp. The appearance of a clear transparent zone was indicative for hydrolysis of the substrate by an extra-cellular protease. The hydrolysis of the tested substrate was developed by overlaying the plates with 15% (w/v) HgCl_2_ in 20% HCl [15]. The diameter of the clear zone was considered a semi-quantitative estimation of protease activity.

### Qualitative screening for keratinase activity

Keratinase activity of each isolate was tested individually by growing in a selective medium with the following composition (g/L): NH_4_Cl 0.5, NaCl 0.5, KH_2_PO_4_ 0.4, K_2_HPO_4_ 0.3, MgCl_2_.6H_2_O 0.1 containing 1% (w/v) feather, under the previously used conditions (Temp. and pH). The degradation of feathers is indicative for hydrolysis of the substrate by an extra-cellular keratinase.

### Effect of pH and Temp. on the growth and enzyme activity

In this experiment, the growth of the selected YNDH strain was evaluated with respect to variation in pH (7–10.5) and Temp. (37–60 °C), using dry weight/OD as a measurable indicator for growth. Also, pH variation (7–10.5) and Temp. (37–70 °C) were tested in order to find the optimal for enzyme activity.

### Quantitative estimation of protease activity

Protease activity was measured by the method of Kembhavi and Kulkarni [16] using 1% casein as a substrate at pH 10 (100 mM glycine-NaOH buffer) and 60 °C (optimal conditions). The absorbance of the soluble fraction was estimated at 280 nm against the blank. A standard curve was generated using L-tyrosine. One unit of protease activity was defined as the amount of enzyme required to liberate 1 μg of tyrosine per min under the experimental conditions.

### Microscopic examination

Phase-contrast microscope (PCM) (AXIOSTAR-plus, ZEISS) was used for morphological characterization of YNDH isolate and Gram stain examination. SEM was performed with JSM 5300 scanning electron microscope (JEOL, USA) at 20 kV in the Centre Laboratory, City of Scientific Research and Technological Applications.

### DNA extraction and PCR for sequencing of 16srRNA PCR product

Salting out method was applied for the total genomic DNA isolation [17] and then used as a template for polymerase chain reaction (PCR). The 16srRNA-PCR product (1.5 kb) was amplified by utilizing gene-specific 16s degenerate primers. TA cloning for the resulting PCR product was performed using InsTAclone PCR Cloning Kit (Fermentas, USA). Automated DNA sequencing for cloned fragment was carried out using the ABI PRISM model 3730 [18]. The extracted purified plasmid was sequenced using M13pUC forward/reverse (F: 5′-GTAAAACGACGGCCAGT-3′ and R: 5′-CAGGAAACAGCTATGAC-3′), and 16srRNA primer and an intermediate forward primer (F: 5′ AGCGGCACCTGAAACTGGAT-3′).

### Alignment and phylogeny

The obtained 16srRNA gene sequences were assembled using DNA STAR program http://www.dnastar.com and BLAST https://blast.ncbi.nlm.nih.gov/Blast.cgi was used to assess the similarity of the assembled sequences. Multiple sequence alignment, molecular phylogeny was performed using MEGA software version 4.0.2 [19]. Subsequently, the sequence has been deposited in the GenBank under accession number MH894395.

### Statistical optimization for production of protease with keratinolytic activity

The optimization of physicochemical factors for a protease with keratinolytic activity production was carried out in two stages. The first was the screening of physicochemical factors using PBD. The second was the optimization of the most significant factors that control the enzyme production process using Box-Behnken design (BBD).

### Plackett-Burman Design (PBD)

The design was used to select the key factors that significantly influenced the protease activity. PB experimental design consisting of a set of 16 experiments (trials) was used to determine the relative significance of 14 factors (variables) that influenced protease production by YNDH in submerged fermentation. PBD depends on the first-order model *Y* = *β*_0_ + ∑ *β*_*i*_*x*_*i*_, where in this model, *Y* represents the response, *β*_0_ is the model intercept, *β*_*i*_ is the variable estimate, and *x*_*i*_ represents the variable. The significance of variables was determined by calculating the *p* value through standard regression analysis. Table 1 illustrates the factors under investigation as well as the levels of each studied factor in the experimental design, and the measured response (protease activity U/ml). For each factor, a high (+ 1) and low (− 1) concentration was tested. All trials were performed in triplicate (using 50 ml medium in a 250 ml Erlenmeyer flasks), and the average value was calculated for the measured response.

**Table 1:** PB experimental design for evaluating factors influencing on protease with keratinolytic activity production by *Laceyella sacchari* NYDH

Trials	NH_4_CL, *X*_1_	Feather, *X*_2_	KH_2_PO_4_, *X*_3_	K_2_HPO_4_, *X*_4_	Yeast extract, *X*_5_	NaCl, *X*_6_	MgSO_4_.7H_2_O, *X*_7_	CaCl_2_. *X*_8_	CuSO_4_, *X*_9_	MnSO_4_, *X*_10_	NaNO_3_, *X*_11_	Temp. (°C), *X*_12_	pH, *X*_13_	Inoculum size, *X*_14_	Activity*
1	1 (0.1)	1 (1)	− 1 (0.01)	− 1 (0.01)	1 (0.05)	1 (0.1)	1 (0.02)	− 1 (0)	− 1 (0)	− 1 (0)	1 (0.02)	− 1 (45)	1 (8)	1 (5%)	1146.2
2	1 (0.1)	− 1 (0.2)	− 1 (0.01)	1 (0.05)	1 (0.05)	1 (0.1)	− 1 (0)	− 1 (0)	− 1 (0)	1 (0.02)	− 1 (0)	1 (55)	1 (8)	− 1 (1%)	106.82
3	− 1 (0.02)	− 1 (0.2)	1 (1)	1 (0.05)	1 (0.05)	− 1 (0)	− 1 (0)	− 1 (0)	1 (0.02)	− 1 (0)	1 (0.02)	1 (55)	− 1 (6)	1 (5%)	51.02
4	− 1 (0.02)	1 (1)	1 (1)	1 (0.05)	− 1 (0)	− 1 (0)	− 1 (0)	1 (0.02)	− 1 (0)−	1 (0.02)	1 (0.02)	− 1 (45)	1 (8)	1 (5%)	50.62
5	1 (0.1)	1 (1)	1 (1)	− 1 (0.01)	− 1 (0)	− 1 (0)	1 (0.02)	− 1 (0)	1 (0.02)	1 (0.02)	− 1 (0)	1 (55)	1 (8)	− 1 (1%)	34.12
6	1 (0.1)	1 (1)	− 1 (0.01)	− 1 (0.01)	− 1 (0)	1 (0.1)	− 1 (0)	1 (0.02)	1 (0.02)	− 1 (0)	1 (0.02)	1 (55)	− 1 (6)	− 1 (1%)	0.00
7	1 (0.1)	− 1 (0.2)	− 1 (0.01)	− 1 (0.01)	1 (0.05)	− 1 (0)	1 (0.02)	1 (0.02)	− 1 (0)	1 (0.02)	1 (0.02)	− 1 (45)	− 1 (6)	1 (5%)	688.1
8	− 1 (0.02)	− 1 (0.2)	− 1 (0.01)	1 (0.05)	− 1 (0)	1 (0.1)	1 (0.02)	− 1 (0)	1 (0.02)	1 (0.02)	− 1 (0)	− 1 (45)	1 (8)	1 (5%)	32.62
9	− 1 (0.02)	− 1 (0.2)	1 (1)	− 1 (0.01)	1 (0.05)	1 (0.1)	− 1 (0)	1 (0.02)	1 (0.02)	− 1 (0)	− 1 (0)	1 (55)	1 (8)	1 (5%)	38.62
10	− 1 (0.02)	1 (1)	− 1 (0.01)	1 (0.05)	1 (0.05)	− 1 (0)	1 (0.02)	1 (0.02)	− 1 (0)	− 1 (0)	1 (0.02)	1 (55)	1 (8)	− 1 (1%)	312.6
11	1 (0.1)	− 1 (0.2)	1 (1)	1 (0.05)	− 1 (0)	1 (0.1)	1 (0.02)	− 1 (0)	− 1 (0)	1 (0.02)	1 (0.02)	1 (55)	− 1 (6)	− 1 (1%)	0.00
12	− 1 (0.02)	1 (1)	1 (1)	− 1 (0.01)	1 (0.05)	1 (0.1)	− 1 (0)	− 1 (0)	1 (0.02)	1 (0.02)	1 (0.02)	− 1 (45)	− 1 (6)	− 1 (1%)	20.02
13	1 (0.1)	1 (1)	− 1 (0.01)	1 (0.05)	1 (0.05)	− 1 (0)	− 1 (0)	1 (0.02)	1 (0.02)	1 (0.02)	− 1 (0)	− 1 (45)	− 1 (6)	1 (5%)	26.92
14	1 (0.1)	− 1 (0.2)	1 (1)	1 (0.05)	− 1 (0)	− 1 (0)	1 (0.02)	1 (0.02)	1 (0.02)	− 1 (0)	− 1 (0)	− 1 (45)	1 (8)	− 1 (1%)	3.32
15	− 1 (0.02)	1 (1)	1 (1)	− 1 (0.01)	− 1 (0)	1 (0.1)	1 (0.02)	1 (0.02)	− 1 (0)	− 1 (0)	− 1 (0)	1 (55)	− 1 (6)	1 (5%)	42.12
16	− 1 (0.02)	− 1 (0.2)	− 1 (0.01)	− 1 (0.01)	− 1 (0)	− 1 (0)	− 1 (0)	− 1 (0)	− 1 (0)	− 1 (0)	− 1 (0)	− 1 (45)	− 1 (6)	− 1 (1%)	0.00

− 1 low level and + 1 high level

Levels of independent variables (X_1_–X_14_) presented between brackets are expressed in terms of (%W/v) or value

X_14_—inoculum size of 48 h where 1% OD = 0.08 and 5% OD = 0.4, in case of low level − 1 and high level + 1, respectively.

*Activity (average of three measurements) was measured as protease using casein substrate expressed as U/ml.

### Response surface methodology (RSM) through Box-Behnken Design (BBD)

RSM was used to optimize the screened components for enhanced protease production using BBD. After estimating the relative significance of independent variables, the most significant three variables were selected for further determination of their optimal level with respect to enzyme activity (U/ml) as a response. This optimization process involved three main steps: performing the statistically designed experiments, estimating the coefficients of the structured mathematical model, predicting the response, and checking the adequacy of the model [20]. Table 3 represents the design matrix (consisting of 14 trials with two central points), three levels (high, medium, and low) for the selected variables which were denoted by + 1, 0, and − 1, respectively, and the measured response [21]. The created model was applied using the coefficient results of each variable [22]. For three variables, the following second-order polynomial structured model was used:

$$ Y={\beta}_0+{\beta}_1\left({X}_1\right)+{\beta}_2\left({X}_2\right)+{\beta}_3\left({X}_3\right)+{\beta}_{12}\left({X}_1{X}_2\right)+{\beta}_{13}\left({X}_1{X}_3\right)+{\beta}_{23}\left({X}_2{X}_3\right)+{\beta}_{11}{\left({X}_1\right)}^2+{\beta}_{22}{\left({X}_2\right)}^2+{\beta}_{33}{\left({X}_3\right)}^2 $$

where *Y* is the predicted response; *β*_0_ is the model intercept; *X*_1_, *X*_2_, and *X*_3_ are the independent variables; *β*_1_, *β*_2_, and *β*_3_ are linear coefficients; *β*_12_, *β*_13_, and *β*_23_ are cross-product coefficients; and *β*_11_, *β*_22_, and *β*_33_ are the quadratic coefficients.

### Statistical analysis of data

The enzyme activity data were subjected to multiple linear regressions using the JMP program to estimate the *t*-values, *p* values, and confidence levels expressing the *p* values as a percentage. The significance level (*p* value) was determined using the Student *t* test. The *t* test for any individual effect allows an evaluation of the probability of finding the observed effect purely by chance. If the probability of the variable under test is sufficiently small, it will be accepted. The confidence level is an expression of the *p* value in percent. The optimal value of activity was estimated using the JMP program. The simultaneous effects of the three most significant independent factors on each response were visualized using a three-dimensional graph generated by STATISTICA 5.0 software [20].

### Degradation of synthetic keratin-containing substrate (keratin-azure) by YNDH protease/keratinase

To test the ability of YNDH protease/keratinase to degrade synthetic substrate (keratin-azure), 10 ml of the produced enzyme was added to a flask containing 0.1 g keratin-azure in 100 mM glycine-NaOH buffer, pH 10, and then incubated for 1 h, at 60 °C, where the appearance of blue color is considered an indicator of enzyme action.

### Degradation of natural keratin-containing substrates by YNDH isolate

The ability of YNDH isolates to degrade keratin-containing natural substrate (wool, human hair, and nails) was tested individually by replacing the chicken feather, in the optimized medium, with the tested natural inducer at the same concentration (1%) except the nail which was used at 0.1%. The experiment was carried out in a 250-ml Erlenmeyer flask containing 50 ml optimized medium with the following composition (% w/v): NH_4_Cl, 0.08; feather, 1; yeast extract, 0.04; MgSO_4_.7H_2_O, 0.02; NaNO_3_, 0.016; KH_2_PO_4_, 0.01; K_2_HPO_4_, 0.01; pH, 8; inoculum size, 5%; and cultivation Temp., 45 °C for 48 h/or 15 days. Afterwards, several photographs were taken using SEM to examine the effects of degradation on used natural substrates especially feather and nails.

### Dehairing of goat hides by YNDH protease/keratinase

Fresh goatskin was obtained from a local butcher and incubated in a solution of chloroform and ethanol 90% (ratio, 2:1) for 2 h in order to remove lipids and fats [23]. The skin was then washed with Det. and water to remove impurities and dried in an oven at 60 °C overnight. The skin was kept at 4 °C until further use. Goatskin pieces (4 cm^2^ × 4 cm^2^) were incubated in a beaker containing 10 mL of crude protease/keratinase enzyme then completed to 20 ml glycine-NaOH buffer pH 10 and the beaker was incubated at 37 °C for 1 h. Chemical dehairing was performed with 10% lime and 2% sodium sulfide for 1 h at room Temp. Afterwards, several photographs were taken using SEM to examine the effects of bio- and chemical dehairing on the skin surface and hair.

### De-staining of blood-stained and chocolate-stained fabric

Wash performance of the crude protease/keratinase enzyme of YNDH strain was evaluated by applying blood and chocolate stains on white cotton fabrics, using a modified method [24], clean white cotton test fabric pieces (4 cm^2^ × 4 cm^2^) were stained with chicken blood and chocolate. The stained pieces were allowed to dry. They were incubated in a beaker containing 10 mL of crude protease/keratinase enzyme then completed to 20 mL glycine-NaOH buffer pH 10 and the beaker was incubated at 37 °C for 1 h. A control experiment was conducted under similar conditions, except that no enzyme was added. Stain removal was visually monitored by washing the clothes with tap water.

## Results

### Isolation and screening of protease/keratinase

In a preliminary screening program for isolation of protease producing micro-organisms, 48 isolates were obtained from soil/ water collected samples. Among, 32 isolates showed protease activity by plate assay using gelatin/casein substrate each at the same isolation conditions (pH and Temp.). However, within 32 isolates which showed positive protease activity, only one isolate coded YNDH (isolated from Egyptian El-Minya soil) showed remarkable potency for feather degradation in selective medium (pH 10). In order to determine the optimum conditions required for enzyme activity in cell free supernatant, different pHs and temperatures were tested (data not shown). The maximum enzyme activity (132,9U/ml) was obtained under the optima assay conditions (pH 10 and Temp. 60 °C). On the other hand, the stated optima Temp. and pH for growth of YNDH strain ranged 45–55 °C and pH 8–9 (data not shown).

### Morphological/molecular characteristics of the selected YNDH isolate

**Fig. 1:**
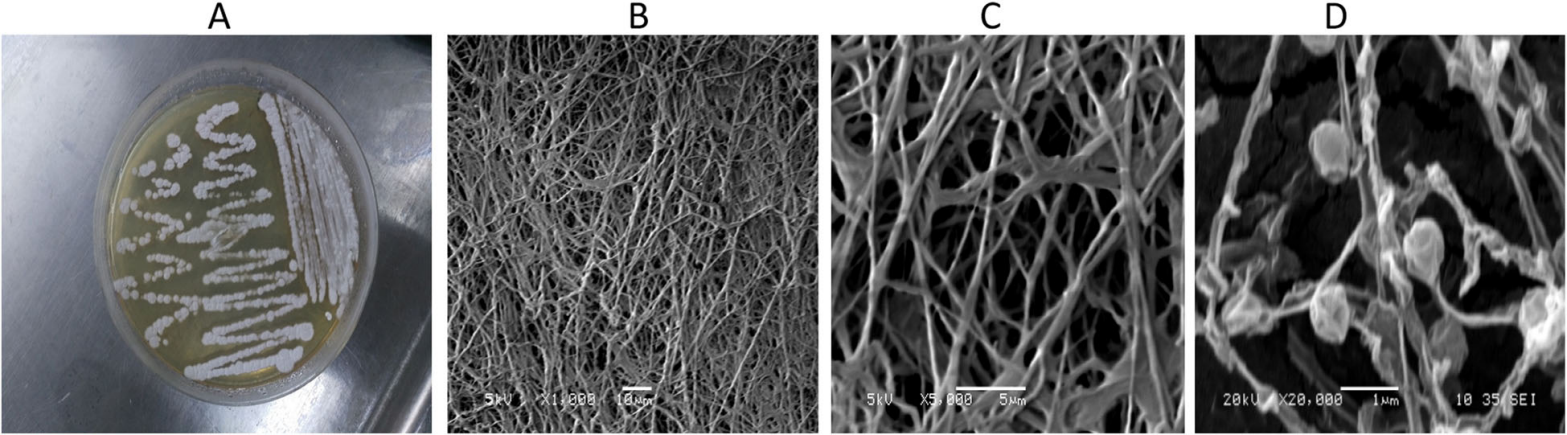
Morphological characteristics of YNDH isolate, where **a** typical growth in a solid medium, **b**, **c** mass of hyphae under phase-contrast microscope at × 1 and × 5 magnification power, respectively, and **d** hyphae and spores under SEM at × 20 magnification power

The isolate is Gram-positive and formed ball-shaped structures in liquid media. A filamentous growth, tangled hyphae, and spores clearly appear under SEM (Fig. 1). The obtained assembled sequence of 16srRNA was submitted to the BLAST in order to find homologies with other relevant sequences, where it showed 99% identity to *Laceyella sacchari* VTT E062990 (ac, EU430566.1). Moreover, the phylogenies of the selected isolate (YNDH) and closely related species was analyzed using the multisequence alignment program and the results confirmed that YNDH was close to *Laceyella sacchari* VTT E062990.

### Statistical optimization for protease production by multi-factorial experiment

PBD design (the first approach) was applied to evaluate the relative significance of cultivation variables affecting the production of protease/keratinase enzyme by *Laceyella sacchari* YNDH strain. Based on the regression analysis shown in Table 2; the regression coefficient of the 14 variables, namely, NH_4_Cl, feather substrate, yeast extract, MgSO_4_, NaNO_3_, pH, and inoculums, had shown a positive effect on protease activity. However, KH_2_PO_4_, NaCl, CaCl_2_, CuSO_4_ MnSO_4_, and cultivation Temp. were found out to contribute negatively. The 14 variables were analyzed using a linear multiple regression analysis method and the % confidence level was calculated on the basis of the confidence level (%) = (1 *− p* value) × 100. Also, the main effect was calculated basically as a difference between the average measurements of each variable made at a high level (+ 1) and a low level (− 1) (Table 2).

**Table 2:** Statistical analysis of PBD showing coefficient values, main effect, *t* and *p* values, and confidence level % for each variable affecting protease with keratinolytic activity production

Variables	Coefficients	Main effect	Standard error	*t* Stat	*p* value	Confidence level (%)
Intercept	159.5688		4.62989	34.4648	0.018466363	
NH_4_Cl	104.7925	209.5849	5.57633	18.7923	0.033844614	96.61554
Feather	15.45379	30.90757	5.35089	2.88807	0.212205337	78.77947
KH_2_PO_4_	− 41.1885	− 82.37706	5.58779	− 7.3711	0.085842193	91.41578
K_2_HPO_4_	− 84.9739	− 169.9478	5.59022	− 15.200	0.04182138	95.81786
Yeast extract	100.5857	201.1713	5.60285	17.9525	0.035424603	96.45754
NaCl	− 6.27788	− 12.55575	5.27715	− 1.1896	0.445003112	55.49969
MgSO_4_.7H_2_O	81.80768	163.6153	5.50027	14.8733	0.042738313	95.72617
CaCl_2_	− 64.1394	− 128.2788	5.50027	− 11.661	0.054460083	94.55399
CuSO_4_	− 79.1459	− 158.2917	5.27715	− 14.9978	0.042384761	95.76152
MnSO_4_	− 77.9249	− 155.817	5.60285	− 13.9081	0.045694769	95.43052
NaNO_3_	95.37533	190.7506	5.59022	17.0610	0.0372715	96.27285
Temp.	− 69.0769	− 138.1537	5.58779	− 12.3621	0.051385815	94.86142
pH	80.68165	161.3632	5.35089	15.0781	0.042159573	95.78404
Inoculum size	99.77608	199.5521	5.57633	17.8927	0.035542747	96.44573

The *p* value from the ANOVA analysis for each response was determined to analyze the relationship between the variables at 90% or higher confidence level. The analysis of variance using ANOVA test gives *p* = 0.0443, indicates that there is a statistically significant relationship between the variables at 95.57% confidence level. The *R*-squared statistic indicates that the model as fitted explains 99.9% of the variability in protease with keratinolytic activity which is the measured response. The polynomial model describing the correlation between the 14 factors and the protease with keratinolytic activity could be presented as follows: *Y* = 159.56875 + 104.7924*X*_1_ + 15.45378*X*_2_ − 41.188533*X*_3_ − 84.973947*X*_4_ + 100.58568*X*_5_ − 6.27787*X*_6_ + 81.807678*X*_7_ − 64.13942*X*_8_ − 79.145864*X*_9_ − 77.924948*X*_10_ + 95.375328*X*_11_ − 69.07686*X*_12_ + 80.681649*X*_13_ + 99.7760828*X*_14_.

From the statistical analysis, great attention was paid to the selected three variables: NH_4_Cl, yeast extract, and NaNO_3_ which significantly affect the protease with keratinolytic activity production, while they have confidence level > 95%. According to these results, a medium with the following composition (%w/v): NH_4_Cl, 0.1; feather, 1; yeast extract, 0.05; MgSO_4_, 0.02; NaNO_3_, 0.02; KH_2_PO_4_, 0.01; K_2_HPO_4_, 0.01; inoculum size 5; pH, 8; Temp. 45 °C; and incubation time 48 h, with enzyme activity 1124 U/ml, was used as the basic medium for the next design.

**Fig. 2:**
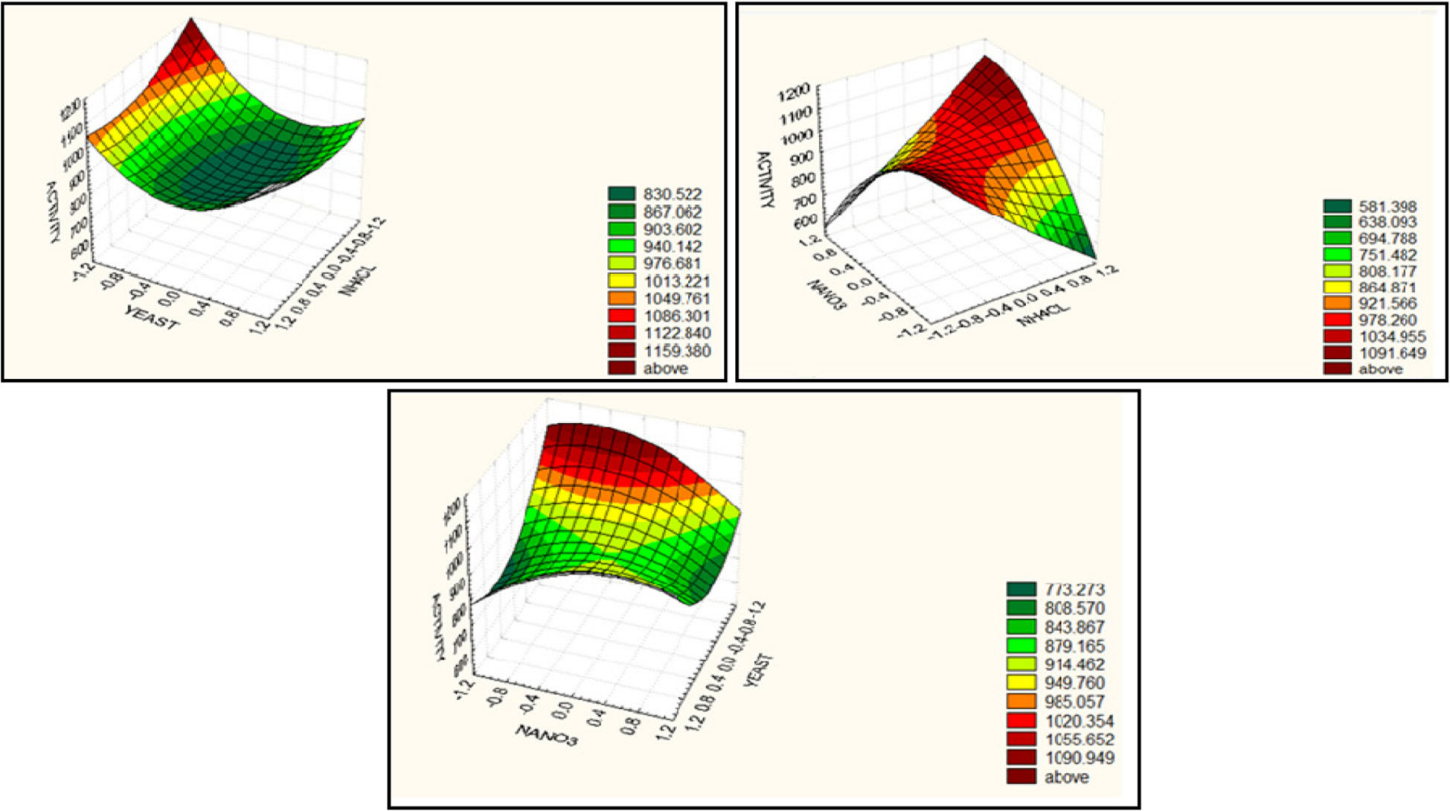
Three-dimensional response surface representing protease with keratinolytic activity yield (U/ml) from *Laceyella sacchari* strain (YNDH) as affected by culture conditions

BBD (the second approach) was applied in order to reach the optimum response region for protease with keratinolytic activity production in term of activity (U/ml), the significant independent variables (*X*_1_; NH_4_Cl, *X*_2_; yeast extract, *X*_3_; NaNO_3_) were further explored, and each at three levels (Table 3). The three variables with fourteen trials were analyzed using a linear multiple regression analysis method, and the percentage confidence levels (%) were calculated as mentioned previously. The value of the determination coefficient *R*^*2*^ = 0.925 for protease with keratinolytic activity, being a measure of fit of the model, indicates that about 7.5% of the total variations are not explained by protease with keratinolytic activity. Presenting experimental results in the form of surface plots shows that higher levels of protease with keratinolytic activity were attained with lower levels of NH_4_Cl, yeast extract, and NaNO_3_ (Fig. 2). For predicting the optimal point of variable, within experimental constrains, a second-order polynomial function was fitted to the experimental results (non-linear optimization algorithm: *Y* = 852.7 − 7.249 *X*_1_ − 45 *X*_2_ − 1.4999 *X*_3_ + 46 *X*_1_*X*_2_ + 210.5 *X*_1_*X*_3_ + 71 *X*_2_*X*_3_ + 37.25 *X*_1_^2^ + 122.25 *X*_2_^2^ − 68.749 *X*_3_^2^).

**Table 3:** Matrix designed, studied variables, and levels for *Laceyella sacchari* strain (YNDH) BB factorial experimental design

Trials	*X*_1_ (NH_4_Cl)	*X*_2_ (yeast extract)	*X*_3_ (NaNO_3_)	*X*_1_*X*_2_	*X*_1_*X*_3_	*X*_2_*X*_3_	*X*_12_	*X*_22_	*X*_32_	Activity*
1	− 1 (0.08)	− 1 (0.04)	0 (0.026)	1	0	0	1	1	0	1139.2
2	1 (0.18)	− 1 (0.04)	0 (0.026)	− 1	0	0	1	1	0	933.2
3	− 1 (0.08)	1 (0.095)	0 (0.026)	− 1	0	0	1	1	0	999.2
4	1 (0.18)	1 (0.095)	0 (0.026)	1	0	0	1	1	0	977.2
5	− 1 (0.08)	0 (0.065)	− 1 (0.016)	0	1	0	1	0	1	1002.2
6	1 (0.18)	0 (0.065)	− 1 (0.016)	0	− 1	0	1	0	1	666.2
7	− 1 (0.08)	0 (0.065)	1 (0.036)	0	− 1	0	1	0	1	555.2
8	1 (0.18)	0 (0.065)	1 (0.036)	0	1	0	1	0	1	1061.2
9	0 (0.13)	− 1 (0.04)	− 1 (0.016)	0	0	1	0	1	1	1033.2
10	0 (0.13)	1 (0.095)	− 1 (0.016)	0	0	− 1	0	1	1	759.2
11	0 (0.13)	− 1 (0.04)	1 (0.036)	0	0	− 1	0	1	1	911.2
12	0 (0.13)	1 (0.095)	1 (0.036)	0	0	1	0	1	1	921.2
13	0 (0.13)	0 (0.065)	0 (0.026)	0	0	0	0	0	0	851.2
14	0 (0.13)	0 (0.065)	0 (0.026)	0	0	0	0	0	0	854.2

Notes: − 1 (low level), 0 (middle level), and + 1 (high level)

Levels of variables (*X*_1_, *X*_2_, and *X*_3_) presented between brackets are expressed in terms of (%w/v)

*Activity (average of three measurements) was measured as protease using casein substrate and expressed as U/ml

The optimal levels of the three studied variables, as obtained from the maximum point of the polynomial model, were found to be (%w/v) NH_4_Cl, 0.08; yeast extract, 0.04; and NaNO_3_, 0.016; with prediction calculated enzyme activity equal to 1324.7 U/ml. Ultimately, in order to determine the accuracy of the quadratic polynomial, a verification experiment was carried out under predicted optimal conditions monitoring enzyme activity in the optimized medium. To prove the accuracy of the model, the % accuracy was calculated from the following formula: Accuracy of the model = [*Y* Experiment/*Y* Calculated] × 100.

The bench-scale experiments show that the *Y* value is 1330.2 U/ml. The calculated model accuracy was 100.4%. In this study, a statistical methodology, a combination of PB and BB designs, showed to be effective and reliable on selecting the statistically significant factors and finding the optimal concentrations of those factors. Accordingly, the following medium composition is expected to be near the optimum (%w/v): NH_4_Cl, 0.08; feather, 1; yeast extract, 0.04; MgSO_4_.7H_2_O, 0.02; NaNO_3_, 0.016; KH_2_PO_4_, 0.01; K_2_HPO_4_, 0.01; pH, 8; inoculum size, 5%; and incubation Temp. 45 °C for 48 h incubation time.

### Utilization/degradation of different keratin-containing substrates and SEM examination

**Fig. 3:**
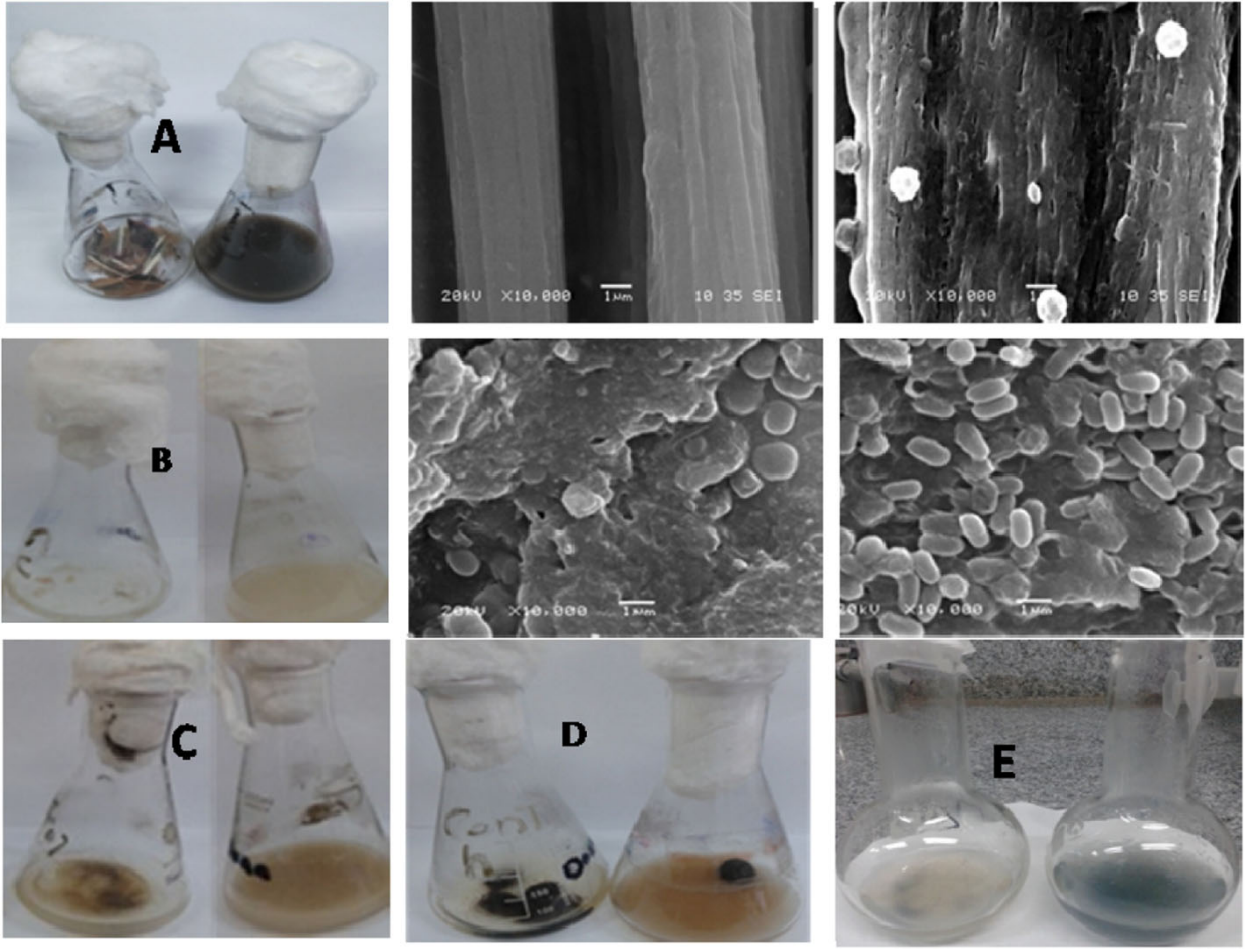
Growth of *Laceyella sacchari* YNDH on feather (**a**), human nails (**b**), and relative SEM examination at × 10 magnification power for control (left) and sample (right). Growth of *Laceyella sacchari* YNDH on wool (**c**), human hair (**d**), and synthetic substrate keratin-azure (**e**), the control at left and the sample at right

In this experiment, we tested the ability of *Laceyella sacchari* YNDH to grow on different substrates (feather, sheep wool, human hair, and human nails), where the degradation of the tested substrate can be easily recognized by eye. Complete degradation of feather and a partial for wool and hair was noticed after 48-h incubation (Fig. 3a–c). A longer time was needed for nail degradation (15 days) as shown in Fig. 3d. Also, by testing the effect of keratinase enzyme produced from YNDH on keratin-azure, the enzyme showed positive reaction indicated by releasing a blue color of azure dye into the buffer (Fig. 3e).

Electron micrographs for degrading feathers and nails were screened using SEM against control. A smooth surface for untreated feather (control) was noticed, while complete disappearance of feather barbs and high degradation of feather rachis during the course of fermentation (48 h) for treated feather were observed (Fig. 3). No degradation for untreated nail (control) under SEM was noticed; however, a high degree of degradation of the nail after 15 days of fermentation was recognized and intensive accumulation of spores on the surface of the degraded nail appeared at high-magnification power examination (Fig. 3).

### De-staining of blood-stained and chocolate-stained fabric

**Fig. 4:**
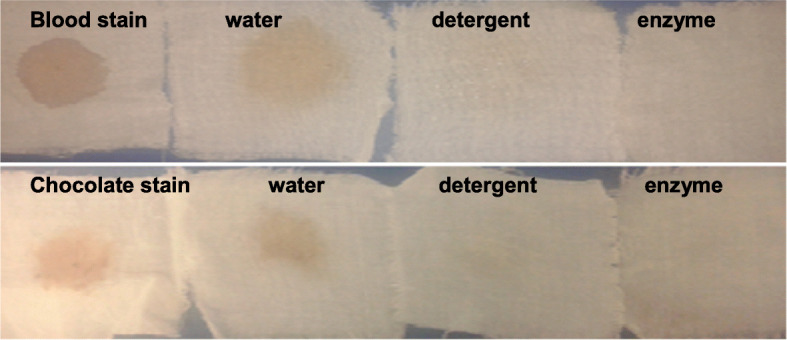
Washing performance test for crude protease/keratinase YNDH in contrast to detergent and water. Stained cloth pieces with blood or chocolate in the left (control: untreated stained cloth pieces). Stained cloth pieces washed with distilled water, Det. (7 mg/ml), and crude enzyme equivalent (20 U/ml)

In order to evaluate the performance of crude enzyme in terms of ability to remove harsh stains, namely those caused by chicken blood or chocolate, several pieces of stained cotton cloth were incubated at different conditions (Fig. 4). The findings from these assays revealed that the blood and chocolate stain removal levels, achieved with the use of crude enzyme alone, were more effective than the ones obtained with Det. or water alone.

### Dehairing of goat hides

**Fig. 5:**
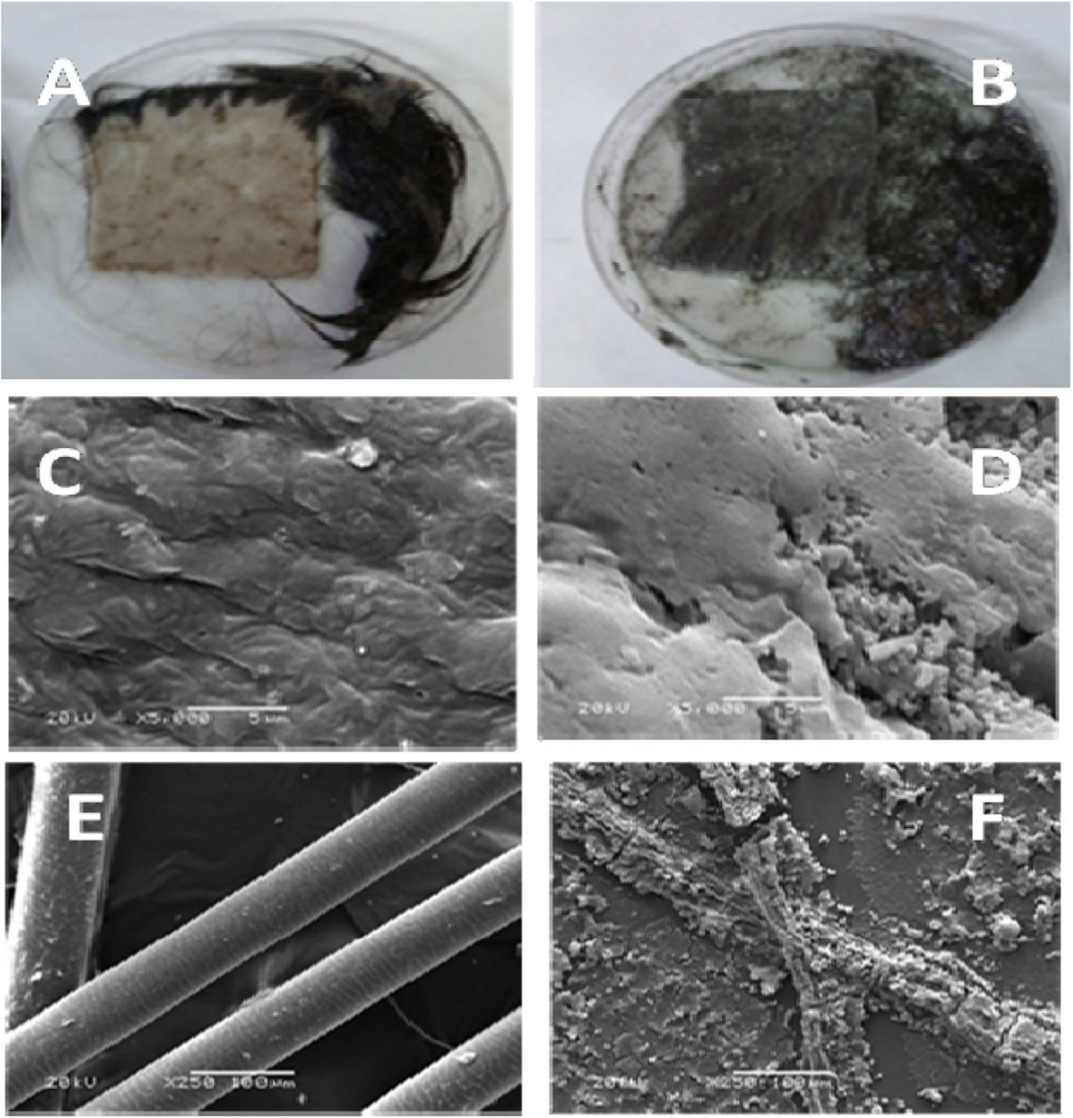
Dehairing efficiency of crude protease/ keratinase (**a**) and lime-sulfide (**b**). SEM examination (× 40) for skin surface (**c**, **d**) and released hairs (**e**, **f**) upon using crude protease/keratinase and lime-sulfide mixture, respectively

The dehairing efficiency for the keratinase enzyme produced from YNDH was tested and examined microscopically. The degree of leather quality generated by the keratinase-based processing was also compared with the conventional chemical treatment of dehairing. The hide surfaces were treated using enzymatic and sulfide lime and the released hairs were examined by scanning electron microscope. The conventional method caused complete degradation of hair and the deposition of the chemicals on the skin surface. In contrast, enzymatic-treated samples caused no degradation of hair and the smooth surface was maintained (Fig. 5a–f).

## Discussion

*Thermoactinomyces* is known for its resistance to extreme environmental conditions and its ability to digest a wide range of hard-to-degrade compounds. Keratinolytic activity has been described for some thermophiles, such as *Thermoactinomyces* spp. [25]. However, this study is considered the first work to report that *Laceyella sacchari* YNDH is a producer of protease/keratinase enzyme, so this strain is distinct in concern its dual activities. Because microbial keratinases are inducible enzymes and are substrate specific [26], various keratinous materials like chicken feathers, feather meal, wool, bovine hair, and human foot skin have been used as an inducer of keratinase [27]. A sequential optimization strategy was implemented in order to improve protease/keratinase production by a locally isolated *Laceyella sacchari* YNDH through two steps (PB and BB). Normally, when screening the variables influencing the development of certain secondary metabolites, it is very essential to test as many variables as possible and to recognize their importance [14]. PB design provides a nice, quick screening process and mathematically calculating the significance of a large number of variables in one experiment that saves time and keeps convincing data about each factor. Although interaction is not included in this model, examining the interaction between these large numbers of factors is not of first priority in the screening program. Only the most efficient and beneficial factors would be chosen from the totality of the variables for further optimization, while those showing a high negative effect on the bioprocess may be dropped in all further experiments. In this study, PB results showed a wide variation from 0 up to 1146.2 U/ml of protease with keratinolytic activity. This variation reflects the importance of medium optimization to attain higher productivity. According to the analysis of the regression coefficients, *t* test, and *p* value for the 14 variables, NH_4_Cl, yeast extract, and NaNO_3_ were the most significant variables increasing the protease with keratinolytic activity production with a *p* value of 0.0338, 0.03542, and 0.03547, respectively, whereas K_2_HPO_4_, MnSO_4_, CuSO_4_, and CaCl_2_ were the most significant variables decreasing the protease with keratinolytic activity production with a *p* value of 0.0418, 0.0456, 0.0423, and 0.054, respectively. This is in agreement with [28, 29], where the yeast extract was found out to exert a positive effect, while CaCO_3_ and, K_2_HPO_4_ had a negative effect on enzyme production by *Amycolatopsis* sp. strain MBRL 40. Additionally, pH showed a significant positive effect on enzyme production while Temp. showed a high negative effect. In general, physical factors such as the optimal Temp. and pH values varied in other researches, depending on the species producing the enzyme [30–34]. One of the advantages of the PBD is that it allows operators to rank the effect of different variables on the measured response independently of the nature of the factor (either nutritional or physical) or the nature of the signs (positive or negative). The second step in the optimization of keratinase production is medium components using statistical methods such as RSM which improve enzyme production. In order to approach the optimum response region of the protease with keratinolytic activity, the significant independent variables (NH_4_Cl, yeast extract, and NaNO_3_) were further studied, each at three levels: − 1, 0 and + 1. Presenting experimental results in the form of surface plots showed that lower levels of yeast extract, NH_4_Cl, and NaNO_3_ support high enzyme production yields/activity. The highest effective interaction was noticed by using the low value of both NH_4_Cl and NaNO_3_. In this experiment, the value of *R*^2^ was 0.925 for protease with keratinolytic activity. This value indicates a high degree of correlation between the experimental and the predicted values. The optimal conditions, realized from the optimization experiment, were verified experimentally and compared to the predicted optimum of the model. The estimated protease with keratinolytic activity was 1330 U/ml, and the predicted value from the polynomial model was 1324.7 U/ml. This high degree of accuracy (100.4%) is evidence of the model validation under optimal conditions. Additionally, the value of enzyme activity in the optimized medium was 10-fold the basal conditions. This reflected the necessity and value of the optimization process. Our work agrees with [35, 36], where the RSM is a widely accepted modern statistical approach for the optimization of the experimental conditions and the solution of the analysis problems, in which a response is greatly influenced by several variables for the production of industrially important biomolecules. RSM helps to identify the successful factors to study interactions, to calculate the optimum level of the variables, and to ensure the maximum production in a fixed number of experiments [36]. Accordingly, the usage of cost-effective growth medium for increasing the yield of proteases with respect to their industrial requirements is highly appreciable from the commercial point of view. In fact, the low-cost substrates were screened for the maximum production of keratinase. Some cost-effective substrates such as soybean meal have been successfully used by Vidyasagar et al. [37]. In conclusion, *Laceyella sacchari* YNDH is a good producer for protease with keratinolytic activity using feathers as the sole source of carbon/nitrogen. In the present work, we examined the effect of *Laceyella sacchari* YNDH on the degradation of feathers, sheep wool, human hair, and human nails, which were significantly degraded. These findings are in agreement with [38], who reported that Keratinases are produced by several bacteria, fungi, and actinomycetes. Due to the ability of *Laceyella sacchari* strain YNDH to significantly degrade/hydrolyze keratin in nails, this opens the way for using YNDH keratinase in pharmaceutical enhancement of the nail treatment. In our work, the findings from Wash performance analysis assays revealed that the blood and chocolate stain removal levels, achieved with the use of crude protease with keratinolytic activity of YNDH alone, were more effective than the ones obtained with Det. alone. In fact, our enzyme facilitated the release of proteinaceous materials in a much easier way than Det. alone. In fact, a similar study has previously reported the usefulness of alkaline protease, namely SAPB, in the blood and chocolate stain removal from cotton cloth [39]. Leather processing industries generate a lot of toxic pollutants, such as sulfide and chromate, which are found to be detrimental to the environment*.* Therefore, dehairing, using a microbial keratinase, is considered an easy alternative [40]. There are many reports of dehairing of goat/bovine skin, employing purified/semi-purified keratinases [41–43]. In the present study, the crude protease with keratinolytic activity from *Laceyella sacchari* strain YNDH completely dehaired goat hides without affecting the skin quality. In contrast, chemical dehairing had shown complete inability to remove of the hair from the goatskin, because parts of the hair were still seen on the skin.

## Conclusion

Proteases are the most widely exploited enzymes in industry. Thermo-alkaliphilic proteases with keratinolytic activity have a wide range of biotechnological applications due to their ability to act at various harsh conditions and degrade a wide range of keratin-containing natural substrates. Thus, this study has focused on the production, optimization, and multifunctional application of keratin-degrading protease enzyme produced by an Egyptian local isolate *Laceyella sacchari* strain YNDH. The theme chosen for the research can be exploited in many industries and various applications.

## Data Availability

All data generated or analyzed during this study are included in this published article.

## Abbreviations

LB: Luria-Bertani
DNA: Deoxyribonucleic acid
RNA: Ribonucleic acid
PCR: Polymerase chain reaction
OVAT: One variable at a time
SEM: Scanning electron microscope
BBD: Box-Behnken design
RSM: Response surface design
PBD: Plackett-Burman Design
Det.: Detergent
Temp.: Temperature
PCM: Phase-contrast microscope
